# Topics and destinations in comments on YouTube tourism videos during the Covid-19 pandemic

**DOI:** 10.1371/journal.pone.0281100

**Published:** 2023-03-02

**Authors:** Orly Carvache-Franco, Mauricio Carvache-Franco, Wilmer Carvache-Franco, Olga Martin-Moreno

**Affiliations:** 1 Facultad de Economía y Empresa, Universidad Católica de Santiago de Guayaquil, Guayaquil, Ecuador; 2 Universidad Espíritu Santo, Samborondón, Ecuador; 3 Facultad de Ciencias Sociales y Humanísticas, Escuela Superior Politécnica del Litoral, ESPOL, Guayaquil, Ecuador; King Saud University, SAUDI ARABIA

## Abstract

This study examines the comments posted on tourism-related YouTube videos during the Covid-19 pandemic to establish sustainable development strategies in destinations. Its objectives were: (i) to identify the topics of discussion, (ii) to establish the perceptions of tourism in a pandemic crisis, and (iii) to identify the destinations mentioned. The data was collected between January and May 2020. 39,225 comments were extracted in different languages and globally through the YouTube API. The data processing was carried out using the word association technique. The results show that the most discussed topics were: “people,” “country,” “tourist,” “place,” “tourism,” “see,” “visit,” “travel,” “covid-19,” “life,” and “live,” which are the focus of the comments made on the perceptions found and represent the attraction factors shown by the videos and the emotions perceived in the comments. The findings show that users’ perceptions are related to risks since the “Covid-19” pandemic is associated with the impact on tourism, people, destinations, and affected countries. The destinations in the comments were: India, Nepal, China, Kerala, France, Thailand, and Europe. The research has theoretical implications concerning tourists’ perceptions of destinations since new perceptions associated with destinations during the pandemic are shown. Such concerns involve tourist safety and work at the destinations. This research has practical implications since, during the pandemic, companies can develop prevention plans. Also, governments could implement sustainable development plans that contain measures so that tourists can make their trips during a pandemic.

## 1. Introduction

A growing number of studies have been focusing on tourism crises and change in recent years. Yet only a few have explicitly investigated health-related crises [1–4]. In 2020, the COVID-19 virus, has triggered an unprecedented global crisis with enormous impacts on our political, social, and economic systems [5, 6].

The Chinese government first reported this virus to WHO on December 31, 2019. WHO declared the pandemic on March 11, 2020 [7].On March 26, 2020, UNWTO [8] announced that the United Nations Specialized Tourism Agency expected international tourist arrivals to decrease by 20% and 30% in 2020 compared to 2019 figures, due to the COVID-19 pandemic. On May 7, 2020, UNWTO [9] announced that international tourism decreased by 22% in the first quarter and could fall by as much as 60%-80% throughout the year. The Covid-19 crisis currently presents the opportunity to reconsider sustainable tourism transformation on a global level [10]. For Yu et al. [11], future studies should examine how the crisis affect the quality and efficiency of tourism companies’ services: performance and image of the destination and mediation of tourist emotions expressed in social networks during a health crisis.

The tourism activity produces a large amount of data, which can be addressed through social networks [12]. In this way, with the development of information and communications technology, people can share their opinions online, creating user-generated content [13]. These comments from social networks can serve as sources to know tourists’ perceptions and impressions [14]. Nationality and culture are critical elements for tourists’ preferences, and with the processing of tourists’ comments on social networks, the preferences of various nationalities and cultures can be understood [15].

In this context, the present study sets the following objectives: (i) to establish the issues that are discussed in user comments on YouTube videos related to tourism during the Covid-19 pandemic, (ii) to establish the perceptions of tourism in a pandemic crisis, and (iii) to establish the destinations mentioned in the comments on the YouTube videos related to tourism during the Covid-19 pandemic.

## 2. Literature review

### 2.1. Tourism in pandemic crisis

The tourism industry is vulnerable to risks, including pandemics, epidemics, crisis, terrorism, or any risk threatening tourist safety [16, 17]. Pandemics and travel relationships are fundamental to understanding health, security, and global change [18]. Consequently, crises can be a trigger for change, but no crisis has so far been a significant transition event in tourism [19].

The 21st century has already experienced four pandemics: SARS in 2002. Avian influenza in 2009, MERS in 2012, and Ebola, which peaked in 2013–14. Risk often restricts travel and has a negative relationship with tourism demand. For example, SARS and the bird flu in Asia [20, 21], swine flu [22], Ebola [23] and MERS [24].

Few reasearchers have studied the crisis of tourism during a pandemic. Yu et al. [11], concerning Covid-2019, established that the key issues identified and discussed in social networks including the perception of tourists risk, which changes dynamically, the effects of the quality of the service of tourism companies during a crisis, quarantine issues in public health, the authenticity of media coverage, and racial discrimination. Similarly, for Nguyen and Coca-Stefaniak [25], there are significant changes in planned travel behaviors after the Covid-2019 pandemic. More specifically, there is a decrease in the intentions to use public transportation and an increased willingness to using private cars. Therefore, the adverse effects of Covid-2019 on tourism can exacerbate income imbalances and harm social equity. As time passes without a vaccine, the adverse effects will intensify, and this can cause a significant delay in economic recovery [26].

Studies on the perception of risk in tourism during a pandemic have been carried out on the tourist or demand side and the resident side of tourist sites or supply [27]. On the risk of a pandemic by tourists or demand, existing studies focus on consumer behavior and mention that tourists perceive risks differently. This risk is associated with some factors such as health and safety. These risks make tourists change their preference for destinations that they believe have a lower level of health and safety risk [28]. On the demand side, some studies have focused on managing tourism crises that affect destinations, response and recovery strategies, and planning practices [29].

### 2.2. YouTube on social media and in tourism

YouTube videos have emerged as a source for research [30]. Social media can facilitate responses from multiple voices from organizations and consumers [31, 32]. Understanding online dialogues helps understand communication during crises [33]. YouTube is a video-based social network. The main reason for watching videos is that it is relaxing entertainment. At the same time, interacting with others leads them to comment on said videos [34]. The comments posted on YouTube videos are interesting and provide information on the public’s reaction to the videos [35]. In this sense, videos with informative content have high validity when large samples are extracted from different regions [36].

YouTube is a website for broad content distribution and viral content sharing [37]. It is possible to find patterns in the themes and differences of gender and feelings while analyzing YouTube comments [38]. The comments on YouTube allow us to know the users’ emotional states [39]. Approximately 60 to 80% of the comments on YouTube from users may contain opinions, which is why it is an important source for obtaining user opinions, which can affect an organization’s reputation [40].

Various studies have used YouTube as a means of researching social media: analysis of user comments on science YouTube channels [41]; analysis of user comments on museums to establish gender differences [35]; analysis of video comments on anti-tourist incidents [42]; analysis of patterns and trends in YouTube video viewing [43]; and analysis of user behavior associated with the comments made on YouTube [44]. Few studies have raised the influence of social media in disasters and pandemics [45], and social media is considered as an important way of expressing oneself after pandemic disasters [46].

YouTube videos about tourist destinations have been used as a tool to communicate identity and brand. YouTube videos about tourist destinations are promotional and commercial videos, generally of an informative nature that communicate the destination’s brand through attraction factors and emotional values [47].

The offer of tourist destinations is of an intangible nature and due to the fact that they cannot be tested before their acquisition, service providers can take advantage of technology and its advances so that marketing managers in this area can give to know their services through audiovisual content, the purpose of audiovisual advertising material is to help destinations create a positive image, improve perceptions and attitudes, as well as influence visiting behavior, that is, in the visit attempt, in the return, sharing, recommending destinations, among other aspects [48].

Specifically, YouTube allows you to measure DCE (Digital consumer engagement), which consists of the level of interaction that a brand can have in a digital environment, through "clicks" on a video, "likes" "dislikes", comments and the fact of sharing the content. Writing a comment requires attention, time, involvement and cognitive resources of the users, so this gives a high indicator of the DCE mentioned above [49].

Given the success of the YouTube platform, companies have found that creating their own channels is an excellent way to achieve a high level of DCE, reach new audiences and convert visits into sales. YouTube makes it possible to characterize how users react to videos, which allows for a quantitative analysis (through the number of likes, clicks, etc.), as well as a qualitative analysis (analysis of the detailed information in the comments) [50].

### 2.3. Crisis communication theory

The crisis communication theories that have been applied to tourism are: (a) Situational crisis communication theory [51, 52] and Networked crisis communication theory [53, 54]. The latter is used for communication in social media using media such as Facebook or YouTube. The literature on communication in crisis in health-related tourism is still scarce [55, 56].

The theory of situational crisis communication suggests that organizations can use various communication strategies during crises. These strategies depend on the type of crisis, the situation of the crisis, and the organization’s responsibility in the crisis [57]. The theory of situational crisis communication tries to establish strategies of responses to crises with positive results for the organization in the public perception of the crisis and its attitude to protecting its reputation and reducing those adverse effects [51, 52].

During crises, response strategies represent the words or terms and the actions that organizations can take during the crisis [51]. In crises when there is a minimum responsibility of the organization, the appropriate communication strategies are instructions and information adjustment, to achieve positive results for organizations such as protecting their reputation and reducing negative effects [57, 58].

The theory of situational crisis communication in tourism was tested in social media. Barbe and Pennington-Gray [54] used Twitter. While Möller et al. [55] and Ki and Nekmat [58] used Facebook. Sandlin and Gracyalny [59] used YouTube to examine comments on public figures.

The theory of situational crisis communication, like the classical theories of crisis communication, focuses on the interaction of the type of crisis and the communication strategy. It does not consider the importance of the medium used in crisis communication in social media that could have a more significant effect than the effects of the type of crisis [57]. In this sense, for Schultz et al. [53], the consumer has different media types in social media in the context of different responses to the crises.

The Networked crisis communication theory considers that crisis communications distributed by social media can provoke different responses, which are affected or impacted by the medium used, the type of crisis, and also by people’s emotions [53, 54]. Therefore, there is a gap in the literature on the effect of crisis communication in social networks from the recipient’s perspective, since social networks can facilitate responses from multiple voices of organizations and the public or consumers [31].

In summary, the literature indicates that tourists associate health risks during pandemics with the quality of service and safety, which makes them change their preferences for destinations with a lower level of risk. However, little literature addresses the perceptions and reactions in user comments about tourism videos on YouTube at the time of the Covid-19 pandemic concerning whether these perceptions denote risks and show preferences in certain destinations. Therefore, in light of this research gap, this study asks the following research questions:

RQ1. Which topics are discussed in comments in tourism YouTube videos during the Covid-19 pandemic?
RQ2. What perceptions do users have about the tourism crisis during the Covid-19 Pandemic?
RQ3. Which destinations are discussed in comments in tourism YouTube videos during the Covid-19 pandemic?

## 3. Methodology

### 3.1. Techniques for data analysis

In recent years, modern techniques have emerged from analyzing social media’s big data, such as association mining or word associations and the technique of analysis of sentiment of comments. This research uses the technique of word associations or association of word content for comments on YouTube videos.

### 3.1.1 Association mining or word associations

The association technique is used to find syntagmatic relationships between terms or words [60]. It is a technique to find patterns in texts processed in large volumes [61]. It is also a technique used to find the knowledge derived from the extraction of previously unknown patterns [62], and it is a quantitative technique to relate words [63]. When it has large volumes of data, this technique is used to classify and explain using existing knowledge or test hypotheses or interrelations between constructs [64].

Through the analysis of tourism data in the network, connections between textual data terms are found [65]. The word associations generally uses a quantitative approach to analyze more extensive text volumes. It helps discover knowledge by increasing the text’s volume to be analyzed [66].

Association mining or word association has been used in tourism to process volumes of online comments from restaurant customers [67], as well as to process online opinions of travelers [68], to process online comments of tourism products [69], to process online comments of restaurants, hotels and attractions [70], to process online comments of the image of a tourist destination [71], to process online comments that serve to analyze the behavior of tourists to market tourist destinations [72], to process comments online on hotel satisfaction factors [73], to compare various online platforms on hotel population comments [74], to process comments online restaurants [75], to process online comments of customer satisfaction in hotels [76], and to predict tourist demand [77].

Language is the most reliable and common way for humans to make their inner thoughts known in a way that other people can understand [49]. Texts are the direct expressions of users’ needs and emotions, so text-based analysis in the tourism sector has the potential to transform the industry, since decision-making is undoubtedly directly influenced by the experiences of trips of other tourists, made in comments on social networks or through blogs [80]. These texts can provide valuable insights for potential tourists and assist them in optimally choosing a destination, as well as exploring travel routes, or they can also help entrepreneurs in the tourism sector to improve their value proposition [78].

In general, "word association" techniques have been used to propose and perform tourism analysis to develop tourism value analysis models, build tourism recommendation systems, create tourism profiles and create policies to supervise tourism markets [78]. The advanced analysis of information provided by customers is a way to acquire, maintain, engage and satisfy their needs in an optimal way. It also allows creating transparency, segmentation of the population, replacement or support in the decision-making of individuals [79]. This is the importance of carrying out data analysis, since through comprehensive evaluation indicators, the analysis can be used to create an offer of personalized intelligent services for both tourists and tourism providers [80].

The analysis of social networks or social media analytics—SMA is considered as the structured and unstructured analysis of data obtained through social networks. Carrying out an SMA includes sentiment analysis and is useful to access and understand the behavior of users, or the group of users more broadly defined as society, especially in uncertain conditions such as the COVID-19 pandemic. An SMA generates new leads through customer experience and operational efficiency [79].

### 3.2. Data collection

The data was collected through the YouTube API using Mozdeh big data text analysis software. 158,578 comments were extracted in different languages from different countries. 39,225 comments corresponding to the Covid-19 pandemic, from January to May 2020, were obtained. These comments met the condition that the video included, in its title or the video description, the term “tourism”.

### 3.3. Data analysis

As part of the processing, the data analysis followed these steps:

First, data cleansing was performed checking and removing duplicate comments to improve the quality of the data to be analyzed.

Second, the word association technique was used to obtain the words associated with the term tourism in the YouTube data collected using Mozdeh big data text analysis software automatically check for associations between a query keywords extracting the words from all matching texts, and further analysing the statistically significant terms with a quantitative process with Pearson’s Chi-square statistical test, derived from a 2x2 contingency table used with a critical threshold value of 3,841. The Benjamini and Hochberg [81] method was used to reduce the risk of falsely believing that a word is significant when examining multiple Chi-square values. This procedure tests all the words simultaneously and shows all the words as results or meaningful terms. This method controls the risk of false positives when running multiple tests. Explanatory tables were prepared with the results.

Third, a dendrogram of clusters was developed to identify the most relevant terms or those that belong to each cluster. Fourth, for each discussion topic, the word association technique was used to obtain the five words associated with each discussion topic found in the collected YouTube data. Lastly, the videos’ comments were analyzed to identify the users’ perceptions related to the five words associated with each discussion topic.

## 4. Results

### 4.1. Topics discussed in the comments of YouTube videos during the Covid-19 pandemic

The data processing using the word association technique is shown in Table 1. The most discussed topics in the YouTube comments were: “people,” “country,” “tourist,” “place,” “tourism,” "see,” "visit,” "travel,” "covid-19,” "life,” "live," and others, those topics reflect the main aspects on which users commented at the time of the Covid-19 pandemic. In Table 1, the “Match” column displays the percentage of comments that contain the word that matches the term Covid-19. The “NoMatch” column displays the percentage of comments that contain the word that does not match the data collected with the search term Covid-19. The “Matches” column is the number of comments that match the word with the term Covid-19. The “Total” column is the number of comments that contain the word. The “DiffPZ” column is the difference in proportion z. The “Sig” column shows if the relationship was significant to the Chisq test performed.

**Table 1:** Most discussed terms in comments to tourism videos.

Word	Match	NoMatch	Matches	Total	DiffPZ	Chisq	Sig (92301 tests)
People	22,10%	4,60%	1892	8738	69,1	4773	***
Country	18,90%	3,30%	1623	6581	70,6	4978,1	***
Tourist	15,40%	2,70%	1317	5316	63,5	4034,7	***
Place	14,80%	3,20%	1269	6056	54,6	2976,5	***
Tourism	12,30%	1,90%	1053	3921	60,1	3616,9	***
See	10,10%	2,30%	863	4335	42,8	1832,7	***
Visit	6,60%	2,60%	562	4524	21,2	448,3	***
Travel	6,10%	0,80%	526	1654	47,7	2277,2	***
Know	6,10%	2,60%	522	4489	18,7	349,8	***
Look	5,60%	1,20%	479	2339	32,5	1054,7	***
World	5,40%	1,80%	459	3178	22,8	517,9	***
Covid-19	5,00%	0,30%	429	1136	14,4	206,3	***
Hotel	4,30%	0,50%	371	1103	41,6	1730,8	***
Live	3,90%	2,10%	331	3541	10,5	110	***
Life	3,60%	0,80%	307	1455	26,6	707,2	***
Culture	3,50%	0,60%	303	1195	30,6	937,1	***
Information	3,40%	0,40%	293	830	38,2	1458,2	***
Money	3,10%	1,10%	263	1937	16	256	***
History	3,00%	0,40%	257	884	31,2	973,6	***
Government	2,90%	0,90%	250	1537	18,9	357,9	***
Tour	2,60%	0,60%	222	1122	21,4	456,9	***
Work	2,60%	1,00%	222	1719	13,8	191,6	***
Food	2,40%	1,10%	210	1846	11,4	130,2	***
Island	2,20%	0,40%	191	769	23,9	570,5	***
Problem	2,00%	0,80%	175	1396	11,8	140	***
Home	2,00%	0,60%	172	1118	14,8	219,2	***
Guide	2,00%	0,60%	168	1067	15	224,5	***
Help	1,70%	0,60%	143	1082	11,4	129,9	***
Experience	1,60%	0,50%	138	923	12,9	165,4	***
Price	1,50%	0,40%	127	691	15,1	228,4	***

From the terms found in Table 1, a dendrogram of clusters was obtained in SPSS software using data from matches column of Table 1 to determine the most hierarchical terms based on the frequency of comments for each term, shows that the terms people, country, tourism, tourist, place, see are the most significant in the hierarchy.

**Fig 1:**
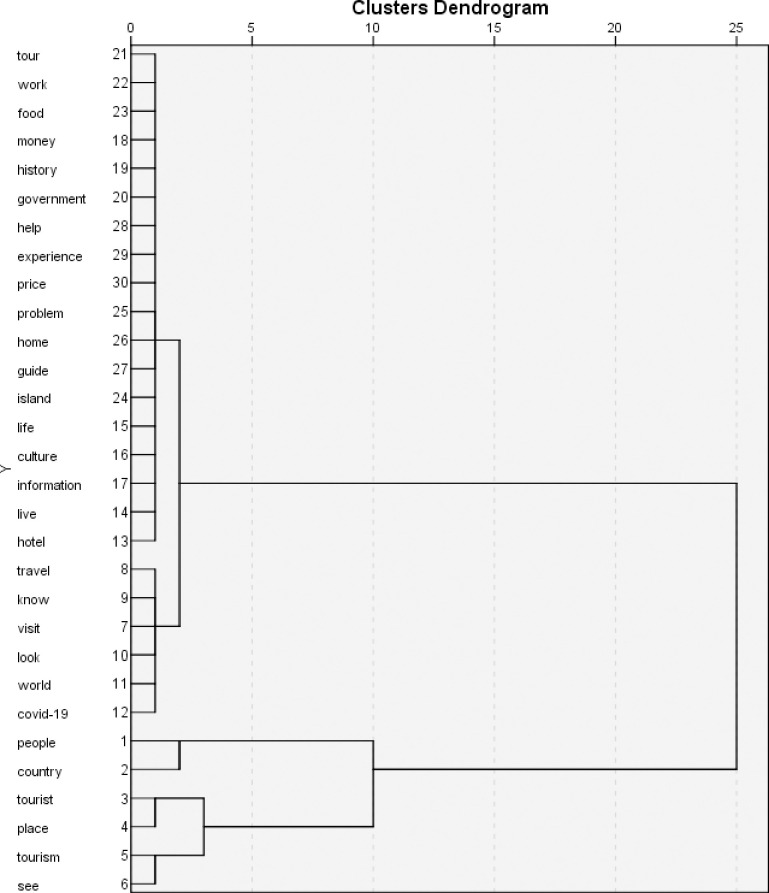
Clusters dendrogram.

It is observed that in cluster 1, there are the terms “people,” “country,” “tourist,” "place," "tourism," and "see" identified as the group with the highest hierarchy (Fig 1).

The results show the topics that users had discussed most frequently in YouTube tourism videos. These topics reflect the main aspects on which users commented at the time of the Covid-19 pandemic: the terms that are the focus of attention of the comments. The findings are shown in Table 1. These results respond to RQ1: Which topics are discussed in comments in tourism YouTube videos during the Covid-19 pandemic? The study found that the most commented topics are mainly: people, country, tourist, place, tourism, see, visit, travel, and others.

The results of YouTube users’ perceptions of the tourism crisis during the pandemic are shown in Table 2 show the perception of users about the situation of destinations, people, tourism, tourists during the pandemic. These respond to RQ2. What perceptions do users have about the tourism crisis during the Covid-2019 Pandemic? The findings show the perceptions grouped into three aspects:

1. The perceptions and impressions of users of tourism videos on YouTube during the Covid-19 pandemic indicate that tourists relate destinations to countries, their people, and the things they can see in those destinations.
2. The term "Covid-19" is associated with the effect or impact on tourism, people, destinations, and countries affected.
3. The tourist perceives the need for work from the destination and the problems they could have in those places.

**Table 2:** The five words associated with each discussion topic and the perceptions in the comments to tourism videos for each discussion topic.

Word	Top 5 terms	Perceptions
People	Like, country, good, want, come	What they like about the people of a country
Country	People, like, about, beautiful, because	Reasons for visiting a country
Tourist	People, from, like, place, country	What tourists like about a country and destinations
Place	Like, people, from, beautiful, tourist	What tourists like about a destination
Tourism	Tourist, people, from, about, country	The tourism of a country, its people, and the origin of the tourist
See	People, there, like, video, place	What do you like to see about people and what is in the destination? Watch video.
Visit	Place, people, tourist, there, country	Reasons why the tourist visits the tourist destination/country and what is there
Travel	People, like, video, there, from	What do people who travel like about travel videos?
Know	That, people, about, what, like	Likes to know about people
Look	Like, people, there, from, about	What do you like to look at the place and about the people
World	People, country, from, tourism, like	Tourism in the world people and countries
Covid-19	People, country, place, there, from	The people, destinations, and countries affected by the Covid-19 pandemic
Hotel	There, tourist, people, like, from	The hotels they like in destinations and the types of tourists in hotels
Live	People, there, tourist, from, country	The people who live there and where they come from
Life	Like, people, from, there, good	The life of the people there
Culture	People, about, from, there, place	The culture of the people and the destination
Information	Good, video, about, guide, nice	The video is a useful guide
Money	People, have, tourist, from, there	The money that people and tourists need in a destination
History	About, video, guide, people, place	Video, place, and people related to history, guide.
Government	People, tourist, country, tourism, about	The government’s relationship with tourism and people
Tour	There, like, have, people, tourist	The tours that tourists like in the place
Work	For, people, have, there, like	Work for the people and work from the tourist destination
Food	For, people, have, there, place	Food for the people at the destination
Island	There, like, tourist, people, tourism	Island likes tourists and people
Problem	For, people, tourist, there, more	The problems that the tourist could have in the destination
Home	For, have, that, people, there	For the house and house of the people there
Guide	For, video, history, about, information	The video is a useful guide and information
Help	For, that, people, have, can	Help for the people. We can help
Experience	People, have, with, there, tourist	People’s experience with the tourist
Price	For, tourist, have, people, there	Prices for tourists at the destination

### 4.2. Destinations discussed in the tourism YouTube videos

The most mentioned destinations in the comments of the YouTube users in the tourism videos are shown in Table 3, these destinations focus on in the perception of users. The answer to the RQ3: Which destinations are discussed in comments in tourism YouTube videos during the Covid-19 pandemic? is given. The most commented destinations were: India, Nepal, China, Kerala, France, and Thailand.

**Table 3:** Most frequent destinations mentioned in the comments of videos about tourism on YouTube.

Word	Match	NoMatch	Matches	Total	DiffPZ	Chisq	Sig (92301 tests)
India	3,20%	1,90%	278	3094	8,9	79	***
Nepal	2,30%	0,50%	195	956	20,6	422,7	***
China	1,90%	1,10%	165	1825	6,9	47,7	***
Kerala	1,90%	1,00%	162	1706	7,5	56,4	***
France	1,80%	1,00%	152	1639	7	48,4	***
Thailand	1,70%	0,60%	142	1060	11,5	133,2	***
Europe	1,40%	0,40%	119	728	13,1	171,2	***
Italy	1,40%	0,50%	117	942	9,5	91,2	***
Greece	1,30%	0,30%	108	507	15,9	251,3	***
Japan	1,10%	0,30%	98	530	13,3	178	***
UK	1,10%	0,40%	93	735	8,7	75,8	***
Spain	1,00%	0,40%	89	637	9,6	91,8	***
Lai	1,00%	0,10%	83	225	20,9	436,7	***
Gambia	0,90%	0,20%	76	372	12,8	164,6	***
Africa	0,90%	0,30%	73	548	8,2	67,4	***
Nepali	0,70%	0,20%	63	292	12,2	149,6	***
Singapore	0,70%	0,20%	63	336	10,8	117,2	***
Venice	0,70%	0,30%	62	522	6,5	42,9	***
Haru	0,70%	0,10%	59	174	16,6	276,7	***
Paris	0,70%	0,20%	58	411	7,8	61,1	***
Asia	0,70%	0,30%	58	463	6,8	46	***
Barcelona	0,50%	0,10%	43	183	10,8	117,3	***
Amsterdam	0,50%	0,20%	42	301	6,6	43,1	***
Hawaii	0,40%	0,10%	38	144	11,1	124,1	***

## 5. Discussion

YouTube videos about tourist destinations are informative and communicate the destination brand through attraction factors and emotional values [47]. Comments on YouTube represent tourists’ perceptions and their impressions [15]. The main discussion topics found in the comments were: "people", "country", "tourist", "place", "tourism", "see", "visit", "travel", "covid-19", " life "," live ", which shows the themes that focus the perceptions and impressions of tourists about the videos and the emotions perceived in the comments.

These perceptions show that tourists relate the destinations with the countries, their people and the things they see in those destinations. Tourists perceive the need for work from the destination and the problems they could have in those places. The findings show that users’ perceptions are related to risks since the "covid-19" pandemic is associated with the impact on tourism, people, destinations, and affected countries. This result is related to other authors’ studies that have found tourists perceive risks in pandemics [11, 28, 29]. Also, with studies on tourism crises that affect destinations [30]. Besides, risk restricts travel and negatively affects tourism demand [11, 21–25]. As a contribution, it is established that these responses incorporate preferences such as tourist safety and work from the destination, which the tourist has associated with the destinations related to the Covid-19 situation, which is concerning other studies that mention significant changes in tourist behavior during pandemics [29] and changes in planned travel behaviors after the covid-19 pandemic [26].

Other terms used were "information" and "guide" that indicate that consumers considered the videos in information and guides about destinations appropriate, and terms such as "hotel," "culture," "history" that showed characteristics of the destinations and "money" what they needed to stay in the destinations. Destinations mentioned in the comments that correspond to consumer perceptions and impressions include: India, Nepal, China, Kerala, France, Thailand and Europe, these destinations were promoted in the tourism videos that tourists focused their reaction on. on the comments.

YouTube videos comprise a type of communication from tourism providers to consumers [47]. However, during tourism crisis caused by the Covid-19 pandemic, these videos available on YouTube, can be considered as a type of crisis communication from organizations to consumers. This crisis communication analyzed under the theory of situational crisis communication, in the case of crises with minimal responsibility of the organizations, corresponds to communicative strategies of adjustment of instructions and information [51, 52] and these YouTube videos related to the tourism are considered informative videos and intended to preserve the reputation of the destination and companies. Analysis of the comments shows that there is a favorable consumer reaction to crisis communication from organizations and destinations related to the countries, their people and the things that can be seen in those destinations. The responses to said consumer crisis communication expressed through comments refer to the impact of the Covid-19 pandemic on the tourist destination, producing an impact on the people, the problems and the work from that place. Tourism service companies using crisis communication strategies through YouTube can preserve the reputation of the destination and the companies.

The responses or crisis communication analyzed through the network crisis communication theory show that videos on YouTube that generally seek to promote tourist destinations can generate various responses driven by the emotions of consumers. Research findings indicate that such responses and reactions range from comments related to people, culture, history, country, the money needed, and the impact of Covid-19 on people and the destination. These being clear problems that can be found in a tourist destination. YouTube, as a medium or social networking, can influence the consumer’s response to the crisis.

As recommendations for the sustainability of destinations in times of health crisis due to the fact that tourists in tourist videos during the pandemic show concern for the people and places affected, it is necessary for governments to take measures in destinations such as: worry that people have work, serve communities in situations of health risk or poverty, worry about the safety of tourists, worry about changes in tourist behavior, collaborate with the planning of tourist trips, improve the stay of tourists in hotels, create tourist packages with biosecurity, and preserve the history and culture of the affected places. For this, all governments could contribute with sustainable plans for tourist trips because they are a source of income that, if properly managed in this sector of the economy, can contribute money and work to the affected areas in times of health crisis and times of pandemics.

## 6. Conclusions

The processing of user comments about tourism videos on YouTube through big data techniques, such as word association, is a suitable means to obtain the perceptions and impressions/reactions of users written in comments to the videos.

The perceptions and impressions of users of tourism videos on YouTube during the Covid-19 pandemic are mainly concentrated on the topics: "people," "country," "tourist," "place," "tourism," "see," evidencing that the tourist in the "Covid-19" pandemic perceives an effect or impact on tourism, people, destinations and affected countries. Interest is shown in the need for work from the destination and tourists’ problems in those places. Destinations mentioned in the comments include India, Nepal, China, Kerala, France, Thailand, and Europe.

This research has theoretical implications regarding the perceptions of tourists about destinations, since the data examined shows new perceptions that tourists have associated with destinations during the Covid-19 pandemic, such as tourist safety and work from destinations, this being the contribution to academic literature.

Comments on tourist videos, analyzed from the situational crisis communication theory [51, 52], show that tourist service companies use crisis communication strategies through videos on YouTube to try to preserve the reputation of destinations. The companies and the comments to the tourist videos, analyzed from the theory of network crisis communication, show the ability of YouTube as a means to influence the response of tourists or consumers in the perception of risk and at the same time show the reactions driven by the emotions of tourists.

This study has practical implications since, during tourism crises caused by pandemics, companies can, through YouTube video, improve business crisis communication strategies and develop pandemic crisis prevention plans. To improve the sustainability of destinations, due to the concern that tourists show in the videos for the people and destinations affected, it is necessary for governments to develop sustainable plans aimed at tourists to generate employment and safety in planned trips so that they contribute to the sustainable development of the affected destinations by conducting responsible and sustainable tourism in times of health crises or pandemics.

Finally, the study’s main limitation is the temporality of the data collected during January and May 2020. As a future research line, it would be interesting to analyse the YouTube data to examine changes in consumer tourist behavior due to the Covid-19 pandemic.
